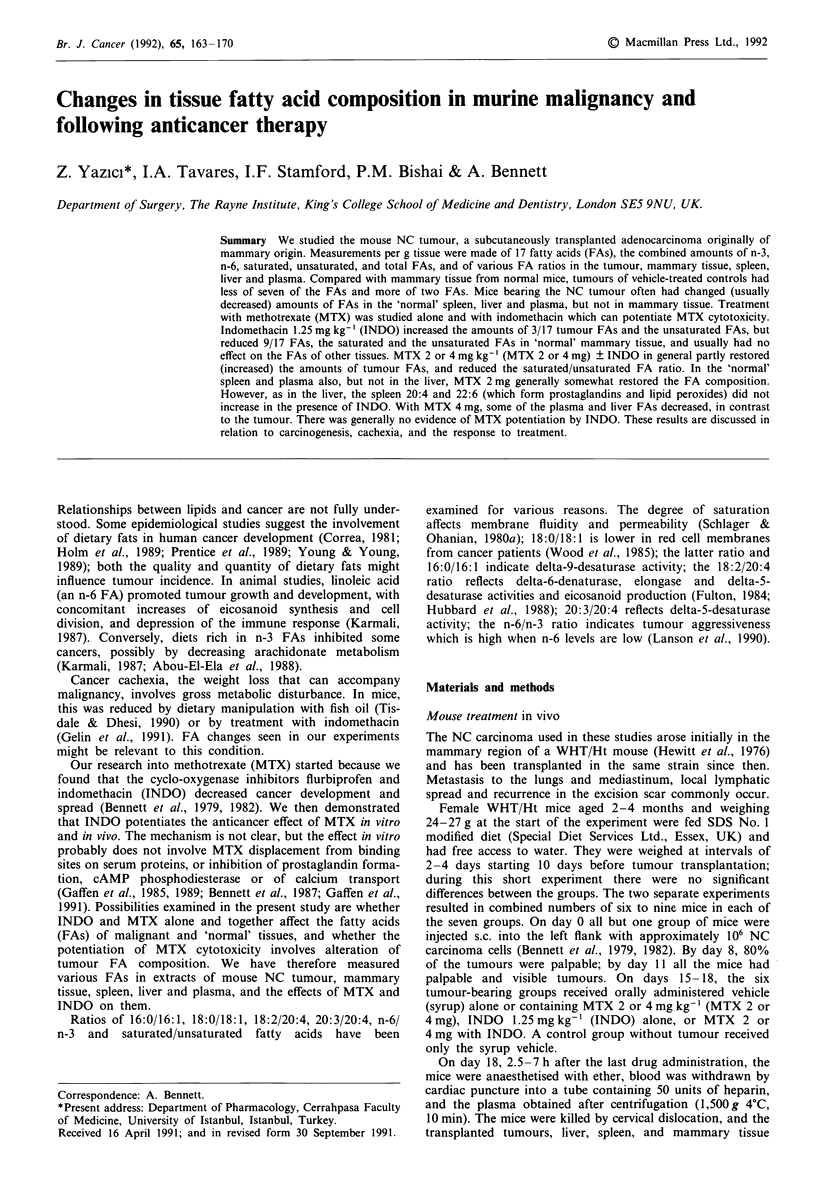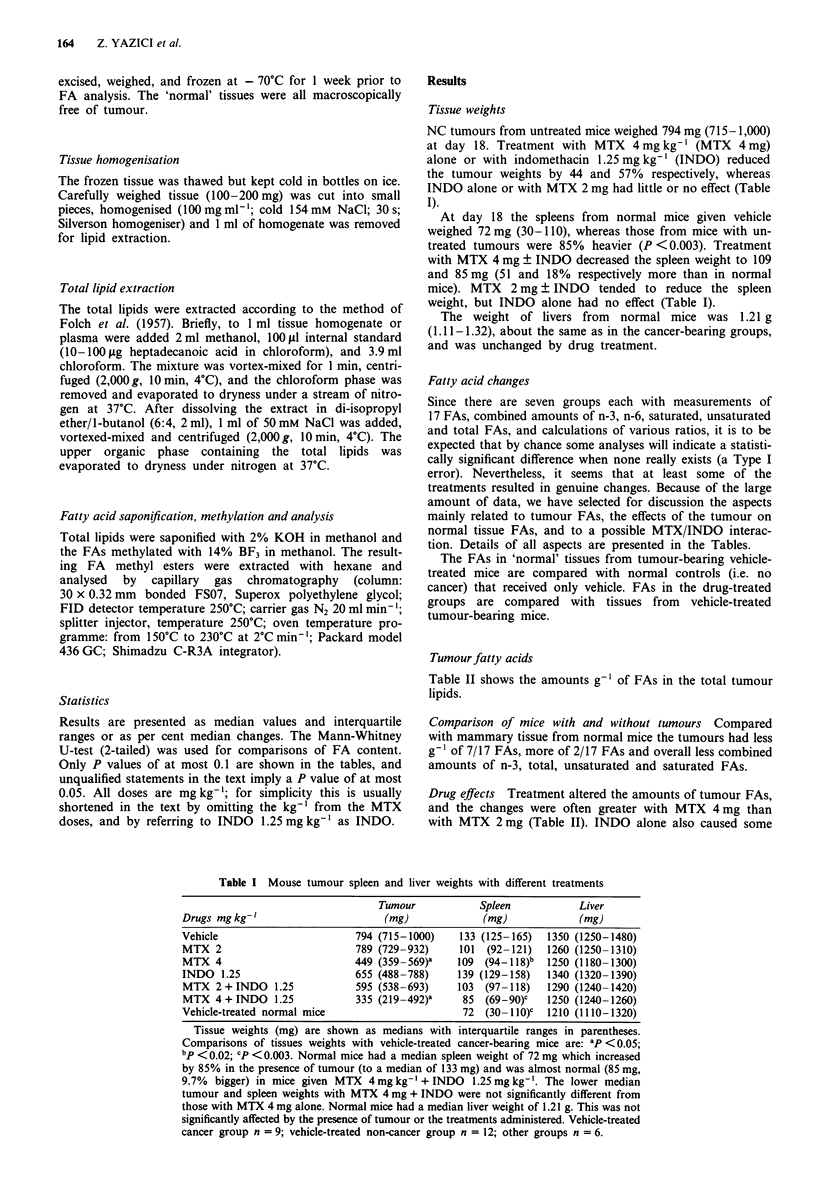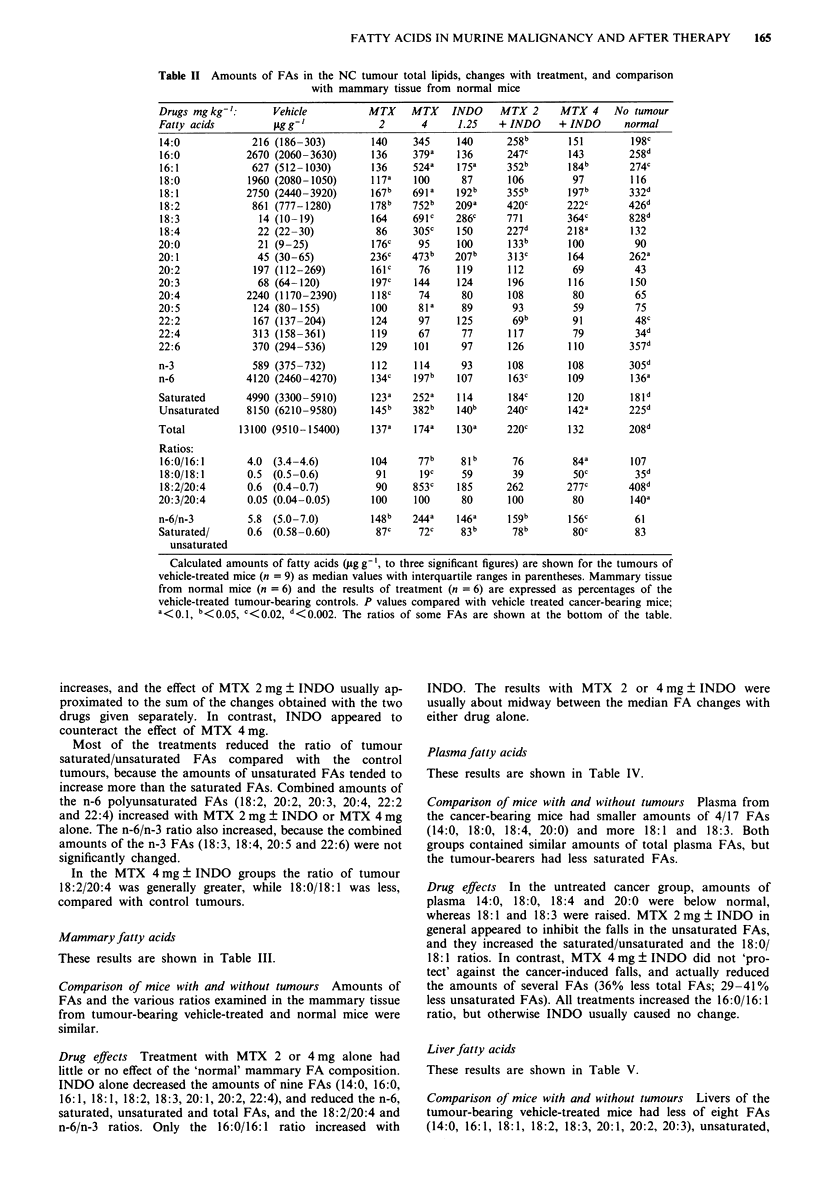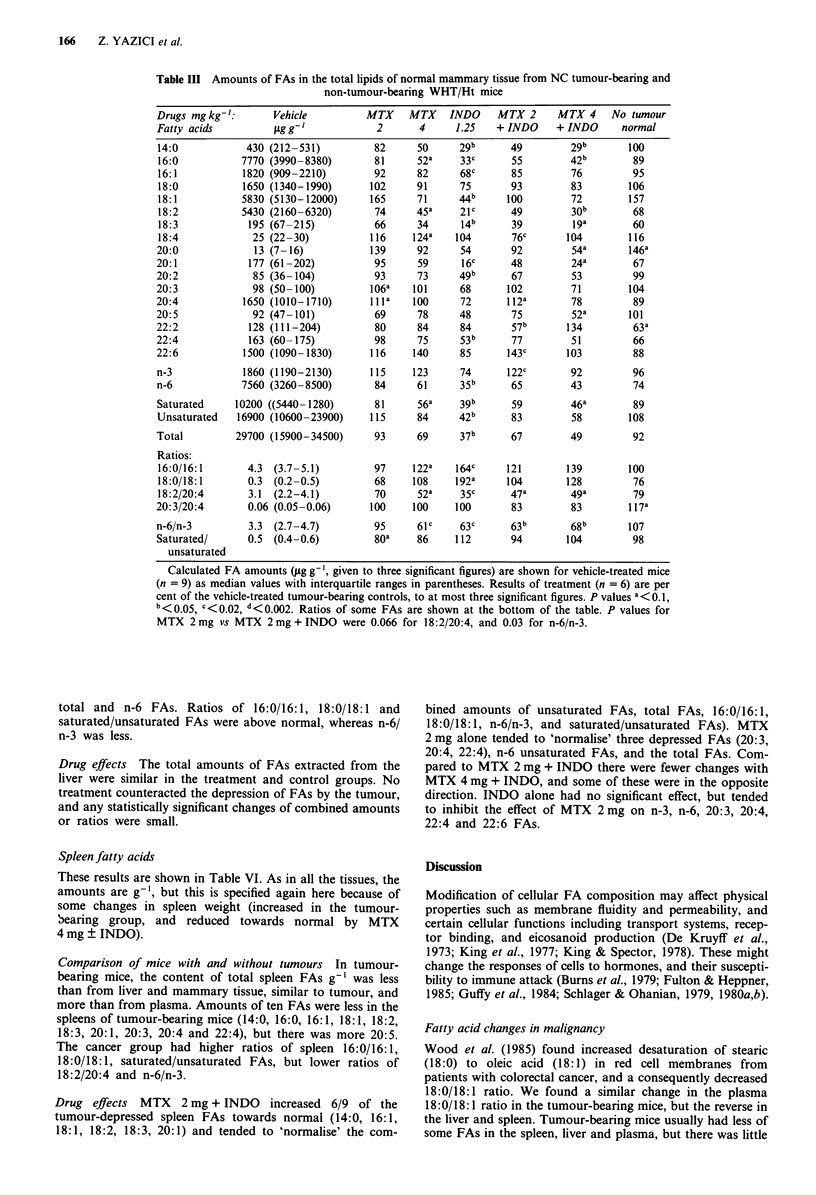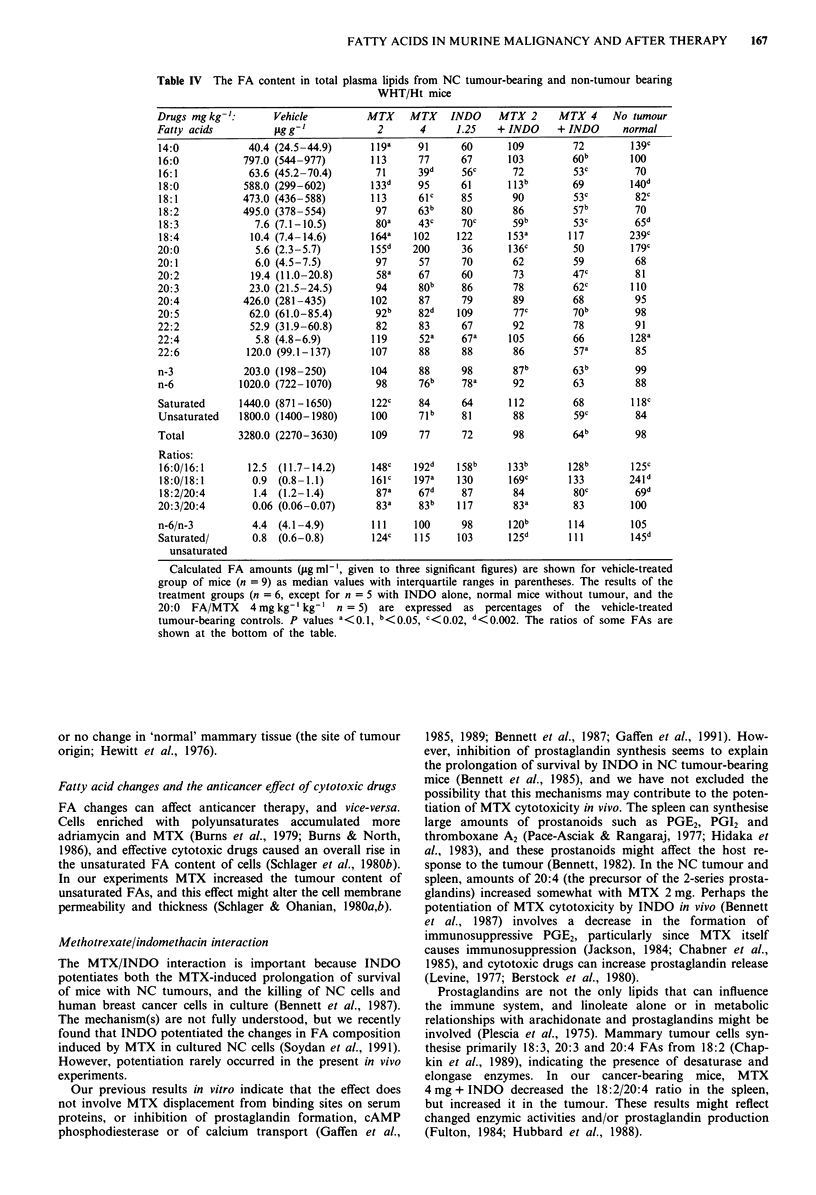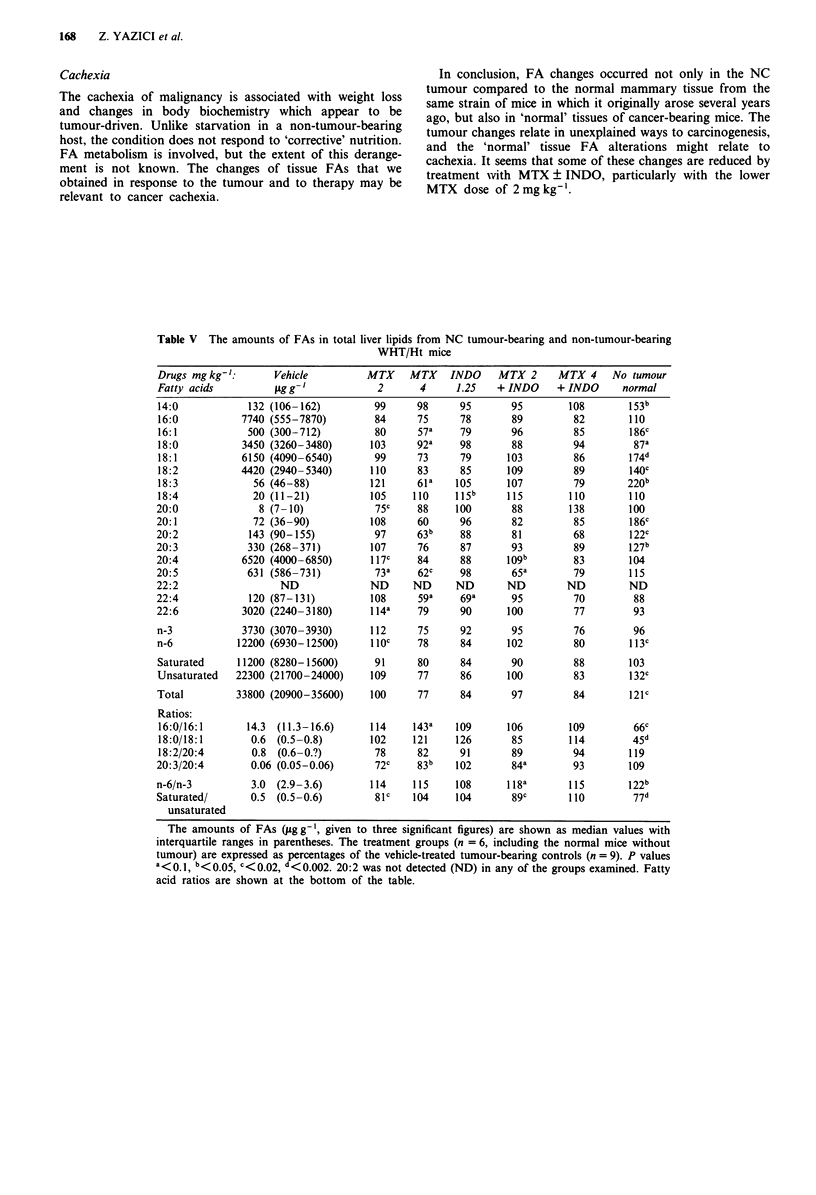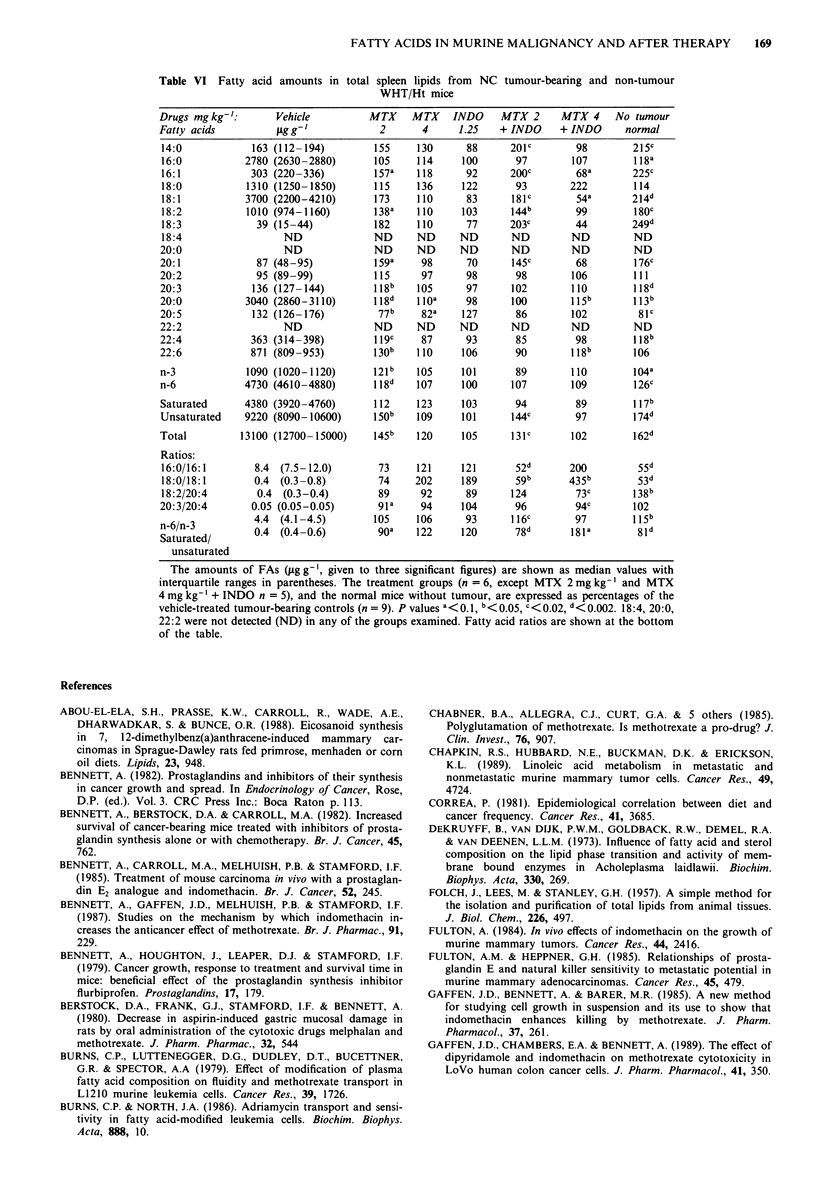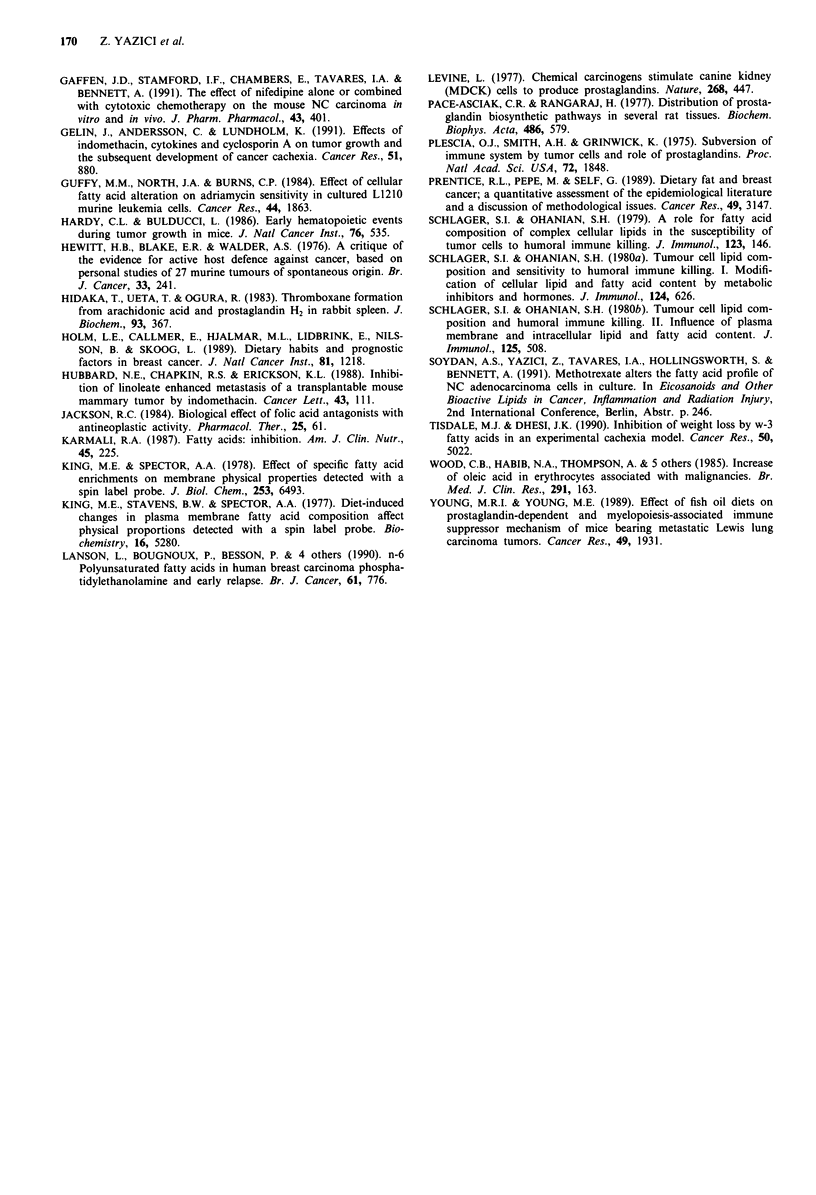# Changes in tissue fatty acid composition in murine malignancy and following anticancer therapy.

**DOI:** 10.1038/bjc.1992.35

**Published:** 1992-02

**Authors:** Z. Yazici, I. A. Tavares, I. F. Stamford, P. M. Bishai, A. Bennett

**Affiliations:** Department of Surgery, Rayne Institute, King's College School of Medicine and Dentistry, London, UK.

## Abstract

We studied the mouse NC tumour, a subcutaneously transplanted adenocarcinoma originally of mammary origin. Measurements per g tissue were made of 17 fatty acids (FAs), the combined amounts of n-3, n-6, saturated, unsaturated, and total FAs, and of various FA ratios in the tumour, mammary tissue, spleen, liver and plasma. Compared with mammary tissue from normal mice, tumours of vehicle-treated controls had less of seven of the FAs and more of two FAs. Mice bearing the NC tumour often had changed (usually decreased) amounts of FAs in the 'normal' spleen, liver and plasma, but not in mammary tissue. Treatment with methotrexate (MTX) was studied alone and with indomethacin which can potentiate MTX cytotoxicity. Indomethacin 1.25 mg kg-1 (INDO) increased the amounts of 3/17 tumours FAs and the unsaturated FAs, but reduced 9/17 FAs, the saturated and the unsaturated FAs in 'normal' mammary tissue, and usually had no effect on the FAs of other tissues. MTX 2 or 4 mg kg-1 (MTX 2 or 4 mg) +/- INDO in general partly restored (increased) the amounts of tumour FAs, and reduced the saturated/unsaturated FA ratio. In the 'normal' spleen and plasma also, but not in the liver, MTX 2 mg generally somewhat restored the FA composition. However, as in the liver, the spleen 20:4 and 22:6 (which form prostaglandins and lipid peroxides) did not increase in the presence of INDO. With MTX 4 mg, some of the plasma and liver FAs decreased, in contrast to the tumour. There was generally no evidence of MTX potentiation by INDO. These results are discussed in relation to carcinogenesis, cachexia, and the response to treatment.


Br. J. Cancer (1992), 65, 163-170 © Macmillan Press Ltd., 1992

### Changes in tissue fatty acid composition in murine malignancy and following anticancer therapy

Z. Yazıcı*, I.A. Tavares, I.F. Stamford, P.M. Bishai & A. Bennett

*Department of Surgery, The Rayne Institute, King's College School of Medicine and Dentistry, London SE5 9NU, UK.*

**Summary** We studied the mouse NC tumour, a subcutaneously transplanted adenocarcinoma originally of mammary origin. Measurements per g tissue were made of 17 fatty acids (FAs), the combined amounts of n-3, n-6, saturated, unsaturated, and total FAs, and of various FA ratios in the tumour, mammary tissue, spleen, liver and plasma. Compared with mammary tissue from normal mice, tumours of vehicle-treated controls had less of seven of the FAs and more of two FAs. Mice bearing the NC tumour often had changed (usually decreased) amounts of FAs in the 'normal' spleen, liver and plasma, but not in mammary tissue. Treatment with methotrexate (MTX) was studied alone and with indomethacin which can potentiate MTX cytotoxicity. Indomethacin 1.25 mg $kg^{-1}$ (INDO) increased the amounts of 3/17 tumour FAs and the unsaturated FAs, but reduced 9/17 FAs, the saturated and the unsaturated FAs in 'normal' mammary tissue, and usually had no effect on the FAs of other tissues. MTX 2 or 4 mg $kg^{-1}$ (MTX 2 or 4 mg) ± INDO in general partly restored (increased) the amounts of tumour FAs, and reduced the saturated/unsaturated FA ratio. In the 'normal' spleen and plasma also, but not in the liver, MTX 2 mg generally somewhat restored the FA composition. However, as in the liver, the spleen 20:4 and 22:6 (which form prostaglandins and lipid peroxides) did not increase in the presence of INDO. With MTX 4 mg, some of the plasma and liver FAs decreased, in contrast to the tumour. There was generally no evidence of MTX potentiation by INDO. These results are discussed in relation to carcinogenesis, cachexia, and the response to treatment.

Relationships between lipids and cancer are not fully understood. Some epidemiological studies suggest the involvement of dietary fats in human cancer development (Correa, 1981; Holm *et al.*, 1989; Prentice *et al.*, 1989; Young & Young, 1989); both the quality and quantity of dietary fats might influence tumour incidence. In animal studies, linoleic acid (an n-6 FA) promoted tumour growth and development, with concomitant increases of eicosanoid synthesis and cell division, and depression of the immune response (Karmali, 1987). Conversely, diets rich in n-3 FAs inhibited some cancers, possibly by decreasing arachidonate metabolism (Karmali, 1987; Abou-El-Ela *et al.*, 1988).

Cancer cachexia, the weight loss that can accompany malignancy, involves gross metabolic disturbance. In mice, this was reduced by dietary manipulation with fish oil (Tisdale & Dhesi, 1990) or by treatment with indomethacin (Gelin *et al.*, 1991). FA changes seen in our experiments might be relevant to this condition.

Our research into methotrexate (MTX) started because we found that the cyclo-oxygenase inhibitors flurbiprofen and indomethacin (INDO) decreased cancer development and spread (Bennett *et al.*, 1979, 1982). We then demonstrated that INDO potentiates the anticancer effect of MTX *in vitro* and *in vivo*. The mechanism is not clear, but the effect *in vitro* probably does not involve MTX displacement from binding sites on serum proteins, or inhibition of prostaglandin formation, cAMP phosphodiesterase or of calcium transport (Gaffen *et al.*, 1985, 1989; Bennett *et al.*, 1987; Gaffen *et al.*, 1991). Possibilities examined in the present study are whether INDO and MTX alone and together affect the fatty acids (FAs) of malignant and 'normal' tissues, and whether the potentiation of MTX cytotoxicity involves alteration of tumour FA composition. We have therefore measured various FAs in extracts of mouse NC tumour, mammary tissue, spleen, liver and plasma, and the effects of MTX and INDO on them.

Ratios of 16:0/16:1, 18:0/18:1, 18:2/20:4, 20:3/20:4, n-6/n-3 and saturated/unsaturated fatty acids have been examined for various reasons. The degree of saturation affects membrane fluidity and permeability (Schlager & Ohanian, 1980*a*); 18:0/18:1 is lower in red cell membranes from cancer patients (Wood *et al.*, 1985); the latter ratio and 16:0/16:1 indicate delta-9-desaturase activity; the 18:2/20:4 ratio reflects delta-6-denaturase, elongase and delta-5-desaturase activities and eicosanoid production (Fulton, 1984; Hubbard *et al.*, 1988); 20:3/20:4 reflects delta-5-desaturase activity; the n-6/n-3 ratio indicates tumour aggressiveness which is high when n-6 levels are low (Lanson *et al.*, 1990).

### Materials and methods

### *Mouse treatment* in vivo

The NC carcinoma used in these studies arose initially in the mammary region of a WHT/Ht mouse (Hewitt *et al.*, 1976) and has been transplanted in the same strain since then. Metastasis to the lungs and mediastinum, local lymphatic spread and recurrence in the excision scar commonly occur.

Female WHT/Ht mice aged 2-4 months and weighing 24-27 g at the start of the experiment were fed SDS No. 1 modified diet (Special Diet Services Ltd., Essex, UK) and had free access to water. They were weighed at intervals of 2-4 days starting 10 days before tumour transplantation; during this short experiment there were no significant differences between the groups. The two separate experiments resulted in combined numbers of six to nine mice in each of the seven groups. On day 0 all but one group of mice were injected s.c. into the left flank with approximately $10^6$ NC carcinoma cells (Bennett *et al.*, 1979, 1982). By day 8, 80% of the tumours were palpable; by day 11 all the mice had palpable and visible tumours. On days 15-18, the six tumour-bearing groups received orally administered vehicle (syrup) alone or containing MTX 2 or 4 mg $kg^{-1}$ (MTX 2 or 4 mg), INDO 1.25 mg $kg^{-1}$ (INDO) alone, or MTX 2 or 4 mg with INDO. A control group without tumour received only the syrup vehicle.

On day 18, 2.5-7 h after the last drug administration, the mice were anaesthetised with ether, blood was withdrawn by cardiac puncture into a tube containing 50 units of heparin, and the plasma obtained after centrifugation (1,500 *g* 4°C, 10 min). The mice were killed by cervical dislocation, and the transplanted tumours, liver, spleen, and mammary tissue

Correspondence: A. Bennett.
*Present address: Department of Pharmacology, Cerrahpasa Faculty of Medicine, University of Istanbul, Istanbul, Turkey.
Received 16 April 1991; and in revised form 30 September 1991.

excised, weighed, and frozen at −70°C for 1 week prior to FA analysis. The 'normal' tissues were all macroscopically free of tumour.

*Tissue homogenisation*

The frozen tissue was thawed but kept cold in bottles on ice. Carefully weighed tissue (100–200 mg) was cut into small pieces, homogenised (100 mg ml$^{-1}$; cold 154 mM NaCl; 30 s; Silverson homogeniser) and 1 ml of homogenate was removed for lipid extraction.

*Total lipid extraction*

The total lipids were extracted according to the method of Folch *et al.* (1957). Briefly, to 1 ml tissue homogenate or plasma were added 2 ml methanol, 100 µl internal standard (10–100 µg heptadecanoic acid in chloroform), and 3.9 ml chloroform. The mixture was vortex-mixed for 1 min, centrifuged (2,000 *g*, 10 min, 4°C), and the chloroform phase was removed and evaporated to dryness under a stream of nitrogen at 37°C. After dissolving the extract in di-isopropyl ether/1-butanol (6:4, 2 ml), 1 ml of 50 mM NaCl was added, vortexed-mixed and centrifuged (2,000 *g*, 10 min, 4°C). The upper organic phase containing the total lipids was evaporated to dryness under nitrogen at 37°C.

*Fatty acid saponification, methylation and analysis*

Total lipids were saponified with 2% KOH in methanol and the FAs methylated with 14% $BF_3$ in methanol. The resulting FA methyl esters were extracted with hexane and analysed by capillary gas chromatography (column: 30 × 0.32 mm bonded FS07, Superox polyethylene glycol; FID detector temperature 250°C; carrier gas $N_2$ 20 ml min$^{-1}$; splitter injector, temperature 250°C; oven temperature programme: from 150°C to 230°C at 2°C min$^{-1}$; Packard model 436 GC; Shimadzu C-R3A integrator).

*Statistics*

Results are presented as median values and interquartile ranges or as per cent median changes. The Mann-Whitney U-test (2-tailed) was used for comparisons of FA content. Only $P$ values of at most 0.1 are shown in the tables, and unqualified statements in the text imply a $P$ value of at most 0.05. All doses are mg kg$^{-1}$; for simplicity this is usually shortened in the text by omitting the kg$^{-1}$ from the MTX doses, and by referring to INDO 1.25 mg kg$^{-1}$ as INDO.

**Results**

*Tissue weights*

NC tumours from untreated mice weighed 794 mg (715–1,000) at day 18. Treatment with MTX 4 mg kg$^{-1}$ (MTX 4 mg) alone or with indomethacin 1.25 mg kg$^{-1}$ (INDO) reduced the tumour weights by 44 and 57% respectively, whereas INDO alone or with MTX 2 mg had little or no effect (Table I).

At day 18 the spleens from normal mice given vehicle weighed 72 mg (30–110), whereas those from mice with untreated tumours were 85% heavier ($P < 0.003$). Treatment with MTX 4 mg ± INDO decreased the spleen weight to 109 and 85 mg (51 and 18% respectively more than in normal mice). MTX 2 mg ± INDO tended to reduce the spleen weight, but INDO alone had no effect (Table I).

The weight of livers from normal mice was 1.21 g (1.11–1.32), about the same as in the cancer-bearing groups, and was unchanged by drug treatment.

*Fatty acid changes*

Since there are seven groups each with measurements of 17 FAs, combined amounts of n-3, n-6, saturated, unsaturated and total FAs, and calculations of various ratios, it is to be expected that by chance some analyses will indicate a statistically significant difference when none really exists (a Type I error). Nevertheless, it seems that at least some of the treatments resulted in genuine changes. Because of the large amount of data, we have selected for discussion the aspects mainly related to tumour FAs, the effects of the tumour on normal tissue FAs, and to a possible MTX/INDO interaction. Details of all aspects are presented in the Tables.

The FAs in 'normal' tissues from tumour-bearing vehicle-treated mice are compared with normal controls (i.e. no cancer) that received only vehicle. FAs in the drug-treated groups are compared with tissues from vehicle-treated tumour-bearing mice.

*Tumour fatty acids*

Table II shows the amounts g$^{-1}$ of FAs in the total tumour lipids.

*Comparison of mice with and without tumours* Compared with mammary tissue from normal mice the tumours had less g$^{-1}$ of 7/17 FAs, more of 2/17 FAs and overall less combined amounts of n-3, total, unsaturated and saturated FAs.

*Drug effects* Treatment altered the amounts of tumour FAs, and the changes were often greater with MTX 4 mg than with MTX 2 mg (Table II). INDO alone also caused some

**Table I** Mouse tumour spleen and liver weights with different treatments

| *Drugs mg kg$^{-1}$* | *Tumour (mg)* | *Spleen (mg)* | *Liver (mg)* |
|---|---|---|---|
| Vehicle | 794 (715–1000) | 133 (125–165) | 1350 (1250–1480) |
| MTX 2 | 789 (729–932) | 101 (92–121) | 1260 (1250–1310) |
| MTX 4 | 449 (359–569)[a] | 109 (94–118)[b] | 1250 (1180–1300) |
| INDO 1.25 | 655 (488–788) | 139 (129–158) | 1340 (1320–1390) |
| MTX 2 + INDO 1.25 | 595 (538–693) | 103 (97–118) | 1290 (1240–1420) |
| MTX 4 + INDO 1.25 | 335 (219–492)[a] | 85 (69–90)[c] | 1250 (1240–1260) |
| Vehicle-treated normal mice | | 72 (30–110)[c] | 1210 (1110–1320) |

Tissue weights (mg) are shown as medians with interquartile ranges in parentheses. Comparisons of tissues weights with vehicle-treated cancer-bearing mice are: [a]$P < 0.05$; [b]$P < 0.02$; [c]$P < 0.003$. Normal mice had a median spleen weight of 72 mg which increased by 85% in the presence of tumour (to a median of 133 mg) and was almost normal (85 mg, 9.7% bigger) in mice given MTX 4 mg kg$^{-1}$ + INDO 1.25 mg kg$^{-1}$. The lower median tumour and spleen weights with MTX 4 mg + INDO were not significantly different from those with MTX 4 mg alone. Normal mice had a median liver weight of 1.21 g. This was not significantly affected by the presence of tumour or the treatments administered. Vehicle-treated cancer group $n = 9$; vehicle-treated non-cancer group $n = 12$; other groups $n = 6$.

**Table II** Amounts of FAs in the NC tumour total lipids, changes with treatment, and comparison with mammary tissue from normal mice

| *Drugs mg kg$^{-1}$: Fatty acids* | *Vehicle μg g$^{-1}$* | *MTX 2* | *MTX 4* | *INDO 1.25* | *MTX 2 + INDO* | *MTX 4 + INDO* | *No tumour normal* |
|---|---|---|---|---|---|---|---|
| 14:0 | 216 (186–303) | 140 | 345 | 140 | 258[b] | 151 | 198[c] |
| 16:0 | 2670 (2060–3630) | 136 | 379[a] | 136 | 247[c] | 143 | 258[d] |
| 16:1 | 627 (512–1030) | 136 | 524[a] | 175[a] | 352[b] | 184[b] | 274[c] |
| 18:0 | 1960 (2080–1050) | 117[a] | 100 | 87 | 106 | 97 | 116 |
| 18:1 | 2750 (2440–3920) | 167[b] | 691[a] | 192[b] | 355[b] | 197[b] | 332[d] |
| 18:2 | 861 (777–1280) | 178[b] | 752[b] | 209[a] | 420[c] | 222[c] | 426[d] |
| 18:3 | 14 (10–19) | 164 | 691[c] | 286[c] | 771 | 364[c] | 828[d] |
| 18:4 | 22 (22–30) | 86 | 305[c] | 150 | 227[d] | 218[a] | 132 |
| 20:0 | 21 (9–25) | 176[c] | 95 | 100 | 133[b] | 100 | 90 |
| 20:1 | 45 (30–65) | 236[c] | 473[b] | 207[b] | 313[c] | 164 | 262[a] |
| 20:2 | 197 (112–269) | 161[c] | 76 | 119 | 112 | 69 | 43 |
| 20:3 | 68 (64–120) | 197[c] | 144 | 124 | 196 | 116 | 150 |
| 20:4 | 2240 (1170–2390) | 118[c] | 74 | 80 | 108 | 80 | 65 |
| 20:5 | 124 (80–155) | 100 | 81[a] | 89 | 93 | 59 | 75 |
| 22:2 | 167 (137–204) | 124 | 97 | 125 | 69[b] | 91 | 48[c] |
| 22:4 | 313 (158–361) | 119 | 67 | 77 | 117 | 79 | 34[d] |
| 22:6 | 370 (294–536) | 129 | 101 | 97 | 126 | 110 | 357[d] |
| n-3 | 589 (375–732) | 112 | 114 | 93 | 108 | 108 | 305[d] |
| n-6 | 4120 (2460–4270) | 134[c] | 197[b] | 107 | 163[c] | 109 | 136[a] |
| Saturated | 4990 (3300–5910) | 123[a] | 252[a] | 114 | 184[c] | 120 | 181[d] |
| Unsaturated | 8150 (6210–9580) | 145[b] | 382[b] | 140[b] | 240[c] | 142[a] | 225[d] |
| Total | 13100 (9510–15400) | 137[a] | 174[a] | 130[a] | 220[c] | 132 | 208[d] |
| Ratios: | | | | | | | |
| 16:0/16:1 | 4.0 (3.4–4.6) | 104 | 77[b] | 81[b] | 76 | 84[a] | 107 |
| 18:0/18:1 | 0.5 (0.5–0.6) | 91 | 19[c] | 59 | 39 | 50[c] | 35[d] |
| 18:2/20:4 | 0.6 (0.4–0.7) | 90 | 853[c] | 185 | 262 | 277[c] | 408[d] |
| 20:3/20:4 | 0.05 (0.04–0.05) | 100 | 100 | 80 | 100 | 80 | 140[a] |
| n-6/n-3 | 5.8 (5.0–7.0) | 148[b] | 244[a] | 146[a] | 159[b] | 156[c] | 61 |
| Saturated/ unsaturated | 0.6 (0.58–0.60) | 87[c] | 72[c] | 83[b] | 78[b] | 80[c] | 83 |

Calculated amounts of fatty acids (μg g$^{-1}$, to three significant figures) are shown for the tumours of vehicle-treated mice ($n = 9$) as median values with interquartile ranges in parentheses. Mammary tissue from normal mice ($n = 6$) and the results of treatment ($n = 6$) are expressed as percentages of the vehicle-treated tumour-bearing controls. *P* values compared with vehicle treated cancer-bearing mice; [a]<0.1, [b]<0.05, [c]<0.02, [d]<0.002. The ratios of some FAs are shown at the bottom of the table.

increases, and the effect of MTX 2 mg ± INDO usually approximated to the sum of the changes obtained with the two drugs given separately. In contrast, INDO appeared to counteract the effect of MTX 4 mg.

Most of the treatments reduced the ratio of tumour saturated/unsaturated FAs compared with the control tumours, because the amounts of unsaturated FAs tended to increase more than the saturated FAs. Combined amounts of the n-6 polyunsaturated FAs (18:2, 20:2, 20:3, 20:4, 22:2 and 22:4) increased with MTX 2 mg ± INDO or MTX 4 mg alone. The n-6/n-3 ratio also increased, because the combined amounts of the n-3 FAs (18:3, 18:4, 20:5 and 22:6) were not significantly changed.

In the MTX 4 mg ± INDO groups the ratio of tumour 18:2/20:4 was generally greater, while 18:0/18:1 was less, compared with control tumours.

### *Mammary fatty acids*

These results are shown in Table III.

*Comparison of mice with and without tumours* Amounts of FAs and the various ratios examined in the mammary tissue from tumour-bearing vehicle-treated and normal mice were similar.

*Drug effects* Treatment with MTX 2 or 4 mg alone had little or no effect of the 'normal' mammary FA composition. INDO alone decreased the amounts of nine FAs (14:0, 16:0, 16:1, 18:1, 18:2, 18:3, 20:1, 20:2, 22:4), and reduced the n-6, saturated, unsaturated and total FAs, and the 18:2/20:4 and n-6/n-3 ratios. Only the 16:0/16:1 ratio increased with INDO. The results with MTX 2 or 4 mg ± INDO were usually about midway between the median FA changes with either drug alone.

### *Plasma fatty acids*

These results are shown in Table IV.

*Comparison of mice with and without tumours* Plasma from the cancer-bearing mice had smaller amounts of 4/17 FAs (14:0, 18:0, 18:4, 20:0) and more 18:1 and 18:3. Both groups contained similar amounts of total plasma FAs, but the tumour-bearers had less saturated FAs.

*Drug effects* In the untreated cancer group, amounts of plasma 14:0, 18:0, 18:4 and 20:0 were below normal, whereas 18:1 and 18:3 were raised. MTX 2 mg ± INDO in general appeared to inhibit the falls in the unsaturated FAs, and they increased the saturated/unsaturated and the 18:0/18:1 ratios. In contrast, MTX 4 mg ± INDO did not 'protect' against the cancer-induced falls, and actually reduced the amounts of several FAs (36% less total FAs; 29–41% less unsaturated FAs). All treatments increased the 16:0/16:1 ratio, but otherwise INDO usually caused no change.

### *Liver fatty acids*

These results are shown in Table V.

*Comparison of mice with and without tumours* Livers of the tumour-bearing vehicle-treated mice had less of eight FAs (14:0, 16:1, 18:1, 18:2, 18:3, 20:1, 20:2, 20:3), unsaturated,

**Table III** Amounts of FAs in the total lipids of normal mammary tissue from NC tumour-bearing and non-tumour-bearing WHT/Ht mice

| *Drugs mg kg$^{-1}$: Fatty acids* | *Vehicle μg g$^{-1}$* | *MTX 2* | *MTX 4* | *INDO 1.25* | *MTX 2 + INDO* | *MTX 4 + INDO* | *No tumour normal* |
|---|---|---|---|---|---|---|---|
| 14:0 | 430 (212–531) | 82 | 50 | 29[b] | 49 | 29[b] | 100 |
| 16:0 | 7770 (3990–8380) | 81 | 52[a] | 33[c] | 55 | 42[b] | 89 |
| 16:1 | 1820 (909–2210) | 92 | 82 | 68[c] | 85 | 76 | 95 |
| 18:0 | 1650 (1340–1990) | 102 | 91 | 75 | 93 | 83 | 106 |
| 18:1 | 5830 (5130–12000) | 165 | 71 | 44[b] | 100 | 72 | 157 |
| 18:2 | 5430 (2160–6320) | 74 | 45[a] | 21[c] | 49 | 30[b] | 68 |
| 18:3 | 195 (67–215) | 66 | 34 | 14[b] | 39 | 19[a] | 60 |
| 18:4 | 25 (22–30) | 116 | 124[a] | 104 | 76[c] | 104 | 116 |
| 20:0 | 13 (7–16) | 139 | 92 | 54 | 92 | 54[a] | 146[a] |
| 20:1 | 177 (61–202) | 95 | 59 | 16[c] | 48 | 24[a] | 67 |
| 20:2 | 85 (36–104) | 93 | 73 | 49[b] | 67 | 53 | 99 |
| 20:3 | 98 (50–100) | 106[a] | 101 | 68 | 102 | 71 | 104 |
| 20:4 | 1650 (1010–1710) | 111[a] | 100 | 72 | 112[a] | 78 | 89 |
| 20:5 | 92 (47–101) | 69 | 78 | 48 | 75 | 52[a] | 101 |
| 22:2 | 128 (111–204) | 80 | 84 | 84 | 57[b] | 134 | 63[a] |
| 22:4 | 163 (60–175) | 98 | 75 | 53[b] | 77 | 51 | 66 |
| 22:6 | 1500 (1090–1830) | 116 | 140 | 85 | 143[c] | 103 | 88 |
| n-3 | 1860 (1190–2130) | 115 | 123 | 74 | 122[c] | 92 | 96 |
| n-6 | 7560 (3260–8500) | 84 | 61 | 35[b] | 65 | 43 | 74 |
| Saturated | 10200 ((5440–1280) | 81 | 56[a] | 39[b] | 59 | 46[a] | 89 |
| Unsaturated | 16900 (10600–23900) | 115 | 84 | 42[b] | 83 | 58 | 108 |
| Total | 29700 (15900–34500) | 93 | 69 | 37[b] | 67 | 49 | 92 |
| Ratios: | | | | | | | |
| 16:0/16:1 | 4.3 (3.7–5.1) | 97 | 122[a] | 164[c] | 121 | 139 | 100 |
| 18:0/18:1 | 0.3 (0.2–0.5) | 68 | 108 | 192[a] | 104 | 128 | 76 |
| 18:2/20:4 | 3.1 (2.2–4.1) | 70 | 52[a] | 35[c] | 47[a] | 49[a] | 79 |
| 20:3/20:4 | 0.06 (0.05–0.06) | 100 | 100 | 100 | 83 | 83 | 117[a] |
| n-6/n-3 | 3.3 (2.7–4.7) | 95 | 61[c] | 63[c] | 63[b] | 68[b] | 107 |
| Saturated/ unsaturated | 0.5 (0.4–0.6) | 80[a] | 86 | 112 | 94 | 104 | 98 |

Calculated FA amounts (μg g$^{-1}$, given to three significant figures) are shown for vehicle-treated mice ($n$ = 9) as median values with interquartile ranges in parentheses. Results of treatment ($n$ = 6) are per cent of the vehicle-treated tumour-bearing controls, to at most three significant figures. $P$ values [a]<0.1, [b]<0.05, [c]<0.02, [d]<0.002. Ratios of some FAs are shown at the bottom of the table. $P$ values for MTX 2 mg *vs* MTX 2 mg + INDO were 0.066 for 18:2/20:4, and 0.03 for n-6/n-3.

total and n-6 FAs. Ratios of 16:0/16:1, 18:0/18:1 and saturated/unsaturated FAs were above normal, whereas n-6/n-3 was less.

*Drug effects* The total amounts of FAs extracted from the liver were similar in the treatment and control groups. No treatment counteracted the depression of FAs by the tumour, and any statistically significant changes of combined amounts or ratios were small.

### *Spleen fatty acids*

These results are shown in Table VI. As in all the tissues, the amounts are g$^{-1}$, but this is specified again here because of some changes in spleen weight (increased in the tumour-bearing group, and reduced towards normal by MTX 4 mg ± INDO).

*Comparison of mice with and without tumours* In tumour-bearing mice, the content of total spleen FAs g$^{-1}$ was less than from liver and mammary tissue, similar to tumour, and more than from plasma. Amounts of ten FAs were less in the spleens of tumour-bearing mice (14:0, 16:0, 16:1, 18:1, 18:2, 18:3, 20:1, 20:3, 20:4 and 22:4), but there was more 20:5. The cancer group had higher ratios of spleen 16:0/16:1, 18:0/18:1, saturated/unsaturated FAs, but lower ratios of 18:2/20:4 and n-6/n-3.

*Drug effects* MTX 2 mg + INDO increased 6/9 of the tumour-depressed spleen FAs towards normal (14:0, 16:1, 18:1, 18:2, 18:3, 20:1) and tended to 'normalise' the combined amounts of unsaturated FAs, total FAs, 16:0/16:1, 18:0/18:1, n-6/n-3, and saturated/unsaturated FAs). MTX 2 mg alone tended to 'normalise' three depressed FAs (20:3, 20:4, 22:4), n-6 unsaturated FAs, and the total FAs. Compared to MTX 2 mg + INDO there were fewer changes with MTX 4 mg + INDO, and some of these were in the opposite direction. INDO alone had no significant effect, but tended to inhibit the effect of MTX 2 mg on n-3, n-6, 20:3, 20:4, 22:4 and 22:6 FAs.

### Discussion

Modification of cellular FA composition may affect physical properties such as membrane fluidity and permeability, and certain cellular functions including transport systems, receptor binding, and eicosanoid production (De Kruyff *et al.*, 1973; King *et al.*, 1977; King & Spector, 1978). These might change the responses of cells to hormones, and their susceptibility to immune attack (Burns *et al.*, 1979; Fulton & Heppner, 1985; Guffy *et al.*, 1984; Schlager & Ohanian, 1979, 1980*a*,*b*).

### *Fatty acid changes in malignancy*

Wood *et al.* (1985) found increased desaturation of stearic (18:0) to oleic acid (18:1) in red cell membranes from patients with colorectal cancer, and a consequently decreased 18:0/18:1 ratio. We found a similar change in the plasma 18:0/18:1 ratio in the tumour-bearing mice, but the reverse in the liver and spleen. Tumour-bearing mice usually had less of some FAs in the spleen, liver and plasma, but there was little

**Table IV** The FA content in total plasma lipids from NC tumour-bearing and non-tumour bearing WHT/Ht mice

| *Drugs mg kg$^{-1}$: Fatty acids* | *Vehicle μg g$^{-1}$* | *MTX 2* | *MTX 4* | *INDO 1.25* | *MTX 2 + INDO* | *MTX 4 + INDO* | *No tumour normal* |
|---|---|---|---|---|---|---|---|
| 14:0 | 40.4 (24.5–44.9) | 119[a] | 91 | 60 | 109 | 72 | 139[c] |
| 16:0 | 797.0 (544–977) | 113 | 77 | 67 | 103 | 60[b] | 100 |
| 16:1 | 63.6 (45.2–70.4) | 71 | 39[d] | 56[c] | 72 | 53[c] | 70 |
| 18:0 | 588.0 (299–602) | 133[d] | 95 | 61 | 113[b] | 69 | 140[d] |
| 18:1 | 473.0 (436–588) | 113 | 61[c] | 85 | 90 | 53[c] | 82[c] |
| 18:2 | 495.0 (378–554) | 97 | 63[b] | 80 | 86 | 57[b] | 70 |
| 18:3 | 7.6 (7.1–10.5) | 80[a] | 43[c] | 70[c] | 59[b] | 53[c] | 65[d] |
| 18:4 | 10.4 (7.4–14.6) | 164[a] | 102 | 122 | 153[a] | 117 | 239[c] |
| 20:0 | 5.6 (2.3–5.7) | 155[d] | 200 | 36 | 136[c] | 50 | 179[c] |
| 20:1 | 6.0 (4.5–7.5) | 97 | 57 | 70 | 62 | 59 | 68 |
| 20:2 | 19.4 (11.0–20.8) | 58[a] | 67 | 60 | 73 | 47[c] | 81 |
| 20:3 | 23.0 (21.5–24.5) | 94 | 80[b] | 86 | 78 | 62[c] | 110 |
| 20:4 | 426.0 (281–435) | 102 | 87 | 79 | 89 | 68 | 95 |
| 20:5 | 62.0 (61.0–85.4) | 92[b] | 82[d] | 109 | 77[c] | 70[b] | 98 |
| 22:2 | 52.9 (31.9–60.8) | 82 | 83 | 67 | 92 | 78 | 91 |
| 22:4 | 5.8 (4.8–6.9) | 119 | 52[a] | 67[a] | 105 | 66 | 128[a] |
| 22:6 | 120.0 (99.1–137) | 107 | 88 | 88 | 86 | 57[a] | 85 |
| n-3 | 203.0 (198–250) | 104 | 88 | 98 | 87[b] | 63[b] | 99 |
| n-6 | 1020.0 (722–1070) | 98 | 76[b] | 78[a] | 92 | 63 | 88 |
| Saturated | 1440.0 (871–1650) | 122[c] | 84 | 64 | 112 | 68 | 118[c] |
| Unsaturated | 1800.0 (1400–1980) | 100 | 71[b] | 81 | 88 | 59[c] | 84 |
| Total | 3280.0 (2270–3630) | 109 | 77 | 72 | 98 | 64[b] | 98 |
| Ratios: | | | | | | | |
| 16:0/16:1 | 12.5 (11.7–14.2) | 148[c] | 192[d] | 158[b] | 133[b] | 128[b] | 125[c] |
| 18:0/18:1 | 0.9 (0.8–1.1) | 161[c] | 197[a] | 130 | 169[c] | 133 | 241[d] |
| 18:2/20:4 | 1.4 (1.2–1.4) | 87[a] | 67[d] | 87 | 84 | 80[c] | 69[d] |
| 20:3/20:4 | 0.06 (0.06–0.07) | 83[a] | 83[b] | 117 | 83[a] | 83 | 100 |
| n-6/n-3 | 4.4 (4.1–4.9) | 111 | 100 | 98 | 120[b] | 114 | 105 |
| Saturated/ unsaturated | 0.8 (0.6–0.8) | 124[c] | 115 | 103 | 125[d] | 111 | 145[d] |

Calculated FA amounts (μg ml$^{-1}$, given to three significant figures) are shown for vehicle-treated group of mice ($n$ = 9) as median values with interquartile ranges in parentheses. The results of the treatment groups ($n$ = 6, except for $n$ = 5 with INDO alone, normal mice without tumour, and the 20:0 FA/MTX 4 mg kg$^{-1}$ kg$^{-1}$ $n$ = 5) are expressed as percentages of the vehicle-treated tumour-bearing controls. $P$ values [a]<0.1, [b]<0.05, [c]<0.02, [d]<0.002. The ratios of some FAs are shown at the bottom of the table.

or no change in 'normal' mammary tissue (the site of tumour origin; Hewitt *et al.*, 1976).

*Fatty acid changes and the anticancer effect of cytotoxic drugs*

FA changes can affect anticancer therapy, and *vice-versa*. Cells enriched with polyunsaturates accumulated more adriamycin and MTX (Burns *et al.*, 1979; Burns & North, 1986), and effective cytotoxic drugs caused an overall rise in the unsaturated FA content of cells (Schlager *et al.*, 1980*b*). In our experiments MTX increased the tumour content of unsaturated FAs, and this effect might alter the cell membrane permeability and thickness (Schlager & Ohanian, 1980*a*,*b*).

*Methotrexate/indomethacin interaction*

The MTX/INDO interaction is important because INDO potentiates both the MTX-induced prolongation of survival of mice with NC tumours, and the killing of NC cells and human breast cancer cells in culture (Bennett *et al.*, 1987). The mechanism(s) are not fully understood, but we recently found that INDO potentiated the changes in FA composition induced by MTX in cultured NC cells (Soydan *et al.*, 1991). However, potentiation rarely occurred in the present *in vivo* experiments.

Our previous results *in vitro* indicate that the effect does not involve MTX displacement from binding sites on serum proteins, or inhibition of prostaglandin formation, cAMP phosphodiesterase or of calcium transport (Gaffen *et al.*, 1985, 1989; Bennett *et al.*, 1987; Gaffen *et al.*, 1991). However, inhibition of prostaglandin synthesis seems to explain the prolongation of survival by INDO in NC tumour-bearing mice (Bennett *et al.*, 1985), and we have not excluded the possibility that this mechanisms may contribute to the potentiation of MTX cytotoxicity *in vivo*. The spleen can synthesise large amounts of prostanoids such as $PGE_2$, $PGI_2$ and thromboxane $A_2$ (Pace-Asciak & Rangaraj, 1977; Hidaka *et al.*, 1983), and these prostanoids might affect the host response to the tumour (Bennett, 1982). In the NC tumour and spleen, amounts of 20:4 (the precursor of the 2-series prostaglandins) increased somewhat with MTX 2 mg. Perhaps the potentiation of MTX cytotoxicity by INDO *in vivo* (Bennett *et al.*, 1987) involves a decrease in the formation of immunosuppressive $PGE_2$, particularly since MTX itself causes immunosuppression (Jackson, 1984; Chabner *et al.*, 1985), and cytotoxic drugs can increase prostaglandin release (Levine, 1977; Berstock *et al.*, 1980).

Prostaglandins are not the only lipids that can influence the immune system, and linoleate alone or in metabolic relationships with arachidonate and prostaglandins might be involved (Plescia *et al.*, 1975). Mammary tumour cells synthesise primarily 18:3, 20:3 and 20:4 FAs from 18:2 (Chapkin *et al.*, 1989), indicating the presence of desaturase and elongase enzymes. In our cancer-bearing mice, MTX 4 mg + INDO decreased the 18:2/20:4 ratio in the spleen, but increased it in the tumour. These results might reflect changed enzymic activities and/or prostaglandin production (Fulton, 1984; Hubbard *et al.*, 1988).

*Cachexia*

The cachexia of malignancy is associated with weight loss and changes in body biochemistry which appear to be tumour-driven. Unlike starvation in a non-tumour-bearing host, the condition does not respond to 'corrective' nutrition. FA metabolism is involved, but the extent of this derangement is not known. The changes of tissue FAs that we obtained in response to the tumour and to therapy may be relevant to cancer cachexia.

In conclusion, FA changes occurred not only in the NC tumour compared to the normal mammary tissue from the same strain of mice in which it originally arose several years ago, but also in 'normal' tissues of cancer-bearing mice. The tumour changes relate in unexplained ways to carcinogenesis, and the 'normal' tissue FA alterations might relate to cachexia. It seems that some of these changes are reduced by treatment with MTX ± INDO, particularly with the lower MTX dose of 2 mg $kg^{-1}$.

**Table V** The amounts of FAs in total liver lipids from NC tumour-bearing and non-tumour-bearing WHT/Ht mice

| *Drugs mg $kg^{-1}$: Fatty acids* | *Vehicle μg $g^{-1}$* | *MTX 2* | *MTX 4* | *INDO 1.25* | *MTX 2 + INDO* | *MTX 4 + INDO* | *No tumour normal* |
|---|---|---|---|---|---|---|---|
| 14:0 | 132 (106–162) | 99 | 98 | 95 | 95 | 108 | 153[b] |
| 16:0 | 7740 (555–7870) | 84 | 75 | 78 | 89 | 82 | 110 |
| 16:1 | 500 (300–712) | 80 | 57[a] | 79 | 96 | 85 | 186[c] |
| 18:0 | 3450 (3260–3480) | 103 | 92[a] | 98 | 88 | 94 | 87[a] |
| 18:1 | 6150 (4090–6540) | 99 | 73 | 79 | 103 | 86 | 174[d] |
| 18:2 | 4420 (2940–5340) | 110 | 83 | 85 | 109 | 89 | 140[c] |
| 18:3 | 56 (46–88) | 121 | 61[a] | 105 | 107 | 79 | 220[b] |
| 18:4 | 20 (11–21) | 105 | 110 | 115[b] | 115 | 110 | 110 |
| 20:0 | 8 (7–10) | 75[c] | 88 | 100 | 88 | 138 | 100 |
| 20:1 | 72 (36–90) | 108 | 60 | 96 | 82 | 85 | 186[c] |
| 20:2 | 143 (90–155) | 97 | 63[b] | 88 | 81 | 68 | 122[c] |
| 20:3 | 330 (268–371) | 107 | 76 | 87 | 93 | 89 | 127[b] |
| 20:4 | 6520 (4000–6850) | 117[c] | 84 | 88 | 109[b] | 83 | 104 |
| 20:5 | 631 (586–731) | 73[a] | 62[c] | 98 | 65[a] | 79 | 115 |
| 22:2 | ND | ND | ND | ND | ND | ND | ND |
| 22:4 | 120 (87–131) | 108 | 59[a] | 69[a] | 95 | 70 | 88 |
| 22:6 | 3020 (2240–3180) | 114[a] | 79 | 90 | 100 | 77 | 93 |
| n-3 | 3730 (3070–3930) | 112 | 75 | 92 | 95 | 76 | 96 |
| n-6 | 12200 (6930–12500) | 110[c] | 78 | 84 | 102 | 80 | 113[c] |
| Saturated | 11200 (8280–15600) | 91 | 80 | 84 | 90 | 88 | 103 |
| Unsaturated | 22300 (21700–24000) | 109 | 77 | 86 | 100 | 83 | 132[c] |
| Total | 33800 (20900–35600) | 100 | 77 | 84 | 97 | 84 | 121[c] |
| Ratios: | | | | | | | |
| 16:0/16:1 | 14.3 (11.3–16.6) | 114 | 143[a] | 109 | 106 | 109 | 66[c] |
| 18:0/18:1 | 0.6 (0.5–0.8) | 102 | 121 | 126 | 85 | 114 | 45[d] |
| 18:2/20:4 | 0.8 (0.6–0.?) | 78 | 82 | 91 | 89 | 94 | 119 |
| 20:3/20:4 | 0.06 (0.05–0.06) | 72[c] | 83[b] | 102 | 84[a] | 93 | 109 |
| n-6/n-3 | 3.0 (2.9–3.6) | 114 | 115 | 108 | 118[a] | 115 | 122[b] |
| Saturated/ unsaturated | 0.5 (0.5–0.6) | 81[c] | 104 | 104 | 89[c] | 110 | 77[d] |

The amounts of FAs (μg $g^{-1}$, given to three significant figures) are shown as median values with interquartile ranges in parentheses. The treatment groups ($n = 6$, including the normal mice without tumour) are expressed as percentages of the vehicle-treated tumour-bearing controls ($n = 9$). $P$ values [a]<0.1, [b]<0.05, [c]<0.02, [d]<0.002. 20:2 was not detected (ND) in any of the groups examined. Fatty acid ratios are shown at the bottom of the table.

**Table VI** Fatty acid amounts in total spleen lipids from NC tumour-bearing and non-tumour WHT/Ht mice

| *Drugs mg kg⁻¹:* *Fatty acids* | *Vehicle* $\mu g\, g^{-1}$ | *MTX 2* | *MTX 4* | *INDO 1.25* | *MTX 2 + INDO* | *MTX 4 + INDO* | *No tumour normal* |
|---|---|---|---|---|---|---|---|
| 14:0 | 163 (112–194) | 155 | 130 | 88 | 201[c] | 98 | 215[c] |
| 16:0 | 2780 (2630–2880) | 105 | 114 | 100 | 97 | 107 | 118[a] |
| 16:1 | 303 (220–336) | 157[a] | 118 | 92 | 200[c] | 68[a] | 225[c] |
| 18:0 | 1310 (1250–1850) | 115 | 136 | 122 | 93 | 222 | 114 |
| 18:1 | 3700 (2200–4210) | 173 | 110 | 83 | 181[c] | 54[a] | 214[d] |
| 18:2 | 1010 (974–1160) | 138[a] | 110 | 103 | 144[b] | 99 | 180[c] |
| 18:3 | 39 (15–44) | 182 | 110 | 77 | 203[c] | 44 | 249[d] |
| 18:4 | ND | ND | ND | ND | ND | ND | ND |
| 20:0 | ND | ND | ND | ND | ND | ND | ND |
| 20:1 | 87 (48–95) | 159[a] | 98 | 70 | 145[c] | 68 | 176[c] |
| 20:2 | 95 (89–99) | 115 | 97 | 98 | 98 | 106 | 111 |
| 20:3 | 136 (127–144) | 118[b] | 105 | 97 | 102 | 110 | 118[d] |
| 20:0 | 3040 (2860–3110) | 118[d] | 110[a] | 98 | 100 | 115[b] | 113[b] |
| 20:5 | 132 (126–176) | 77[b] | 82[a] | 127 | 86 | 102 | 81[c] |
| 22:2 | ND | ND | ND | ND | ND | ND | ND |
| 22:4 | 363 (314–398) | 119[c] | 87 | 93 | 85 | 98 | 118[b] |
| 22:6 | 871 (809–953) | 130[b] | 110 | 106 | 90 | 118[b] | 106 |
| n-3 | 1090 (1020–1120) | 121[b] | 105 | 101 | 89 | 110 | 104[a] |
| n-6 | 4730 (4610–4880) | 118[d] | 107 | 100 | 107 | 109 | 126[c] |
| Saturated | 4380 (3920–4760) | 112 | 123 | 103 | 94 | 89 | 117[b] |
| Unsaturated | 9220 (8090–10600) | 150[b] | 109 | 101 | 144[c] | 97 | 174[d] |
| Total | 13100 (12700–15000) | 145[b] | 120 | 105 | 131[c] | 102 | 162[d] |
| Ratios: | | | | | | | |
| 16:0/16:1 | 8.4 (7.5–12.0) | 73 | 121 | 121 | 52[d] | 200 | 55[d] |
| 18:0/18:1 | 0.4 (0.3–0.8) | 74 | 202 | 189 | 59[b] | 435[b] | 53[d] |
| 18:2/20:4 | 0.4 (0.3–0.4) | 89 | 92 | 89 | 124 | 73[c] | 138[b] |
| 20:3/20:4 | 0.05 (0.05–0.05) | 91[a] | 94 | 104 | 96 | 94[c] | 102 |
| n-6/n-3 | 4.4 (4.1–4.5) | 105 | 106 | 93 | 116[c] | 97 | 115[b] |
| Saturated/ unsaturated | 0.4 (0.4–0.6) | 90[a] | 122 | 120 | 78[d] | 181[a] | 81[d] |

The amounts of FAs ($\mu g\, g^{-1}$, given to three significant figures) are shown as median values with interquartile ranges in parentheses. The treatment groups ($n = 6$, except MTX 2 mg kg⁻¹ and MTX 4 mg kg⁻¹ + INDO $n = 5$), and the normal mice without tumour, are expressed as percentages of the vehicle-treated tumour-bearing controls ($n = 9$). $P$ values [a]$<0.1$, [b]$<0.05$, [c]$<0.02$, [d]$<0.002$. 18:4, 20:0, 22:2 were not detected (ND) in any of the groups examined. Fatty acid ratios are shown at the bottom of the table.

### References

ABOU-EL-ELA, S.H., PRASSE, K.W., CARROLL, R., WADE, A.E., DHARWADKAR, S. & BUNCE, O.R. (1988). Eicosanoid synthesis in 7, 12-dimethylbenz(a)anthracene-induced mammary carcinomas in Sprague-Dawley rats fed primrose, menhaden or corn oil diets. *Lipids*, **23**, 948.

BENNETT, A. (1982). Prostaglandins and inhibitors of their synthesis in cancer growth and spread. In *Endocrinology of Cancer*, Rose, D.P. (ed.). Vol. 3. CRC Press Inc.: Boca Raton p. 113.

BENNETT, A., BERSTOCK, D.A. & CARROLL, M.A. (1982). Increased survival of cancer-bearing mice treated with inhibitors of prostaglandin synthesis alone or with chemotherapy. *Br. J. Cancer*, **45**, 762.

BENNETT, A., CARROLL, M.A., MELHUISH, P.B. & STAMFORD, I.F. (1985). Treatment of mouse carcinoma *in vivo* with a prostaglandin $E_2$ analogue and indomethacin. *Br. J. Cancer*, **52**, 245.

BENNETT, A., GAFFEN, J.D., MELHUISH, P.B. & STAMFORD, I.F. (1987). Studies on the mechanism by which indomethacin increases the anticancer effect of methotrexate. *Br. J. Pharmac.*, **91**, 229.

BENNETT, A., HOUGHTON, J., LEAPER, D.J. & STAMFORD, I.F. (1979). Cancer growth, response to treatment and survival time in mice: beneficial effect of the prostaglandin synthesis inhibitor flurbiprofen. *Prostaglandins*, **17**, 179.

BERSTOCK, D.A., FRANK, G.J., STAMFORD, I.F. & BENNETT, A. (1980). Decrease in aspirin-induced gastric mucosal damage in rats by oral administration of the cytotoxic drugs melphalan and methotrexate. *J. Pharm. Pharmac.*, **32**, 544

BURNS, C.P., LUTTENEGGER, D.G., DUDLEY, D.T., BUCETTNER, G.R. & SPECTOR, A.A (1979). Effect of modification of plasma fatty acid composition on fluidity and methotrexate transport in L1210 murine leukemia cells. *Cancer Res.*, **39**, 1726.

BURNS, C.P. & NORTH, J.A. (1986). Adriamycin transport and sensitivity in fatty acid-modified leukemia cells. *Biochim. Biophys. Acta*, **888**, 10.

CHABNER, B.A., ALLEGRA, C.J., CURT, G.A. & 5 others (1985). Polyglutamation of methotrexate. Is methotrexate a pro-drug? *J. Clin. Invest.*, **76**, 907.

CHAPKIN, R.S., HUBBARD, N.E., BUCKMAN, D.K. & ERICKSON, K.L. (1989). Linoleic acid metabolism in metastatic and nonmetastatic murine mammary tumor cells. *Cancer Res.*, **49**, 4724.

CORREA, P. (1981). Epidemiological correlation between diet and cancer frequency. *Cancer Res.*, **41**, 3685.

DEKRUYFF, B., VAN DIJK, P.W.M., GOLDBACK, R.W., DEMEL, R.A. & VAN DEENEN, L.L.M. (1973). Influence of fatty acid and sterol composition on the lipid phase transition and activity of membrane bound enzymes in Acholeplasma laidlawii. *Biochim. Biophys. Acta*, **330**, 269.

FOLCH, J., LEES, M. & STANLEY, G.H. (1957). A simple method for the isolation and purification of total lipids from animal tissues. *J. Biol. Chem.*, **226**, 497.

FULTON, A. (1984). *In vivo* effects of indomethacin on the growth of murine mammary tumors. *Cancer Res.*, **44**, 2416.

FULTON, A.M. & HEPPNER, G.H. (1985). Relationships of prostaglandin E and natural killer sensitivity to metastatic potential in murine mammary adenocarcinomas. *Cancer Res.*, **45**, 479.

GAFFEN, J.D., BENNETT, A. & BARER, M.R. (1985). A new method for studying cell growth in suspension and its use to show that indomethacin enhances killing by methotrexate. *J. Pharm. Pharmacol.*, **37**, 261.

GAFFEN, J.D., CHAMBERS, E.A. & BENNETT, A. (1989). The effect of dipyridamole and indomethacin on methotrexate cytotoxicity in LoVo human colon cancer cells. *J. Pharm. Pharmacol.*, **41**, 350.

GAFFEN, J.D., STAMFORD, I.F., CHAMBERS, E., TAVARES, I.A. & BENNETT, A. (1991). The effect of nifedipine alone or combined with cytotoxic chemotherapy on the mouse NC carcinoma *in vitro* and *in vivo*. *J. Pharm. Pharmacol.*, **43**, 401.

GELIN, J., ANDERSSON, C. & LUNDHOLM, K. (1991). Effects of indomethacin, cytokines and cyclosporin A on tumor growth and the subsequent development of cancer cachexia. *Cancer Res.*, **51**, 880.

GUFFY, M.M., NORTH, J.A. & BURNS, C.P. (1984). Effect of cellular fatty acid alteration on adriamycin sensitivity in cultured L1210 murine leukemia cells. *Cancer Res.*, **44**, 1863.

HARDY, C.L. & BULDUCCI, L. (1986). Early hematopoietic events during tumor growth in mice. *J. Natl Cancer Inst.*, **76**, 535.

HEWITT, H.B., BLAKE, E.R. & WALDER, A.S. (1976). A critique of the evidence for active host defence against cancer, based on personal studies of 27 murine tumours of spontaneous origin. *Br. J. Cancer*, **33**, 241.

HIDAKA, T., UETA, T. & OGURA, R. (1983). Thromboxane formation from arachidonic acid and prostaglandin $H_2$ in rabbit spleen. *J. Biochem.*, **93**, 367.

HOLM, L.E., CALLMER, E., HJALMAR, M.L., LIDBRINK, E., NILSSON, B. & SKOOG, L. (1989). Dietary habits and prognostic factors in breast cancer. *J. Natl Cancer Inst.*, **81**, 1218.

HUBBARD, N.E., CHAPKIN, R.S. & ERICKSON, K.L. (1988). Inhibition of linoleate enhanced metastasis of a transplantable mouse mammary tumor by indomethacin. *Cancer Lett.*, **43**, 111.

JACKSON, R.C. (1984). Biological effect of folic acid antagonists with antineoplastic activity. *Pharmacol. Ther.*, **25**, 61.

KARMALI, R.A. (1987). Fatty acids: inhibition. *Am. J. Clin. Nutr.*, **45**, 225.

KING, M.E. & SPECTOR, A.A. (1978). Effect of specific fatty acid enrichments on membrane physical properties detected with a spin label probe. *J. Biol. Chem.*, **253**, 6493.

KING, M.E., STAVENS, B.W. & SPECTOR, A.A. (1977). Diet-induced changes in plasma membrane fatty acid composition affect physical proportions detected with a spin label probe. *Biochemistry*, **16**, 5280.

LANSON, L., BOUGNOUX, P., BESSON, P. & 4 others (1990). n-6 Polyunsaturated fatty acids in human breast carcinoma phosphatidylethanolamine and early relapse. *Br. J. Cancer*, **61**, 776.

LEVINE, L. (1977). Chemical carcinogens stimulate canine kidney (MDCK) cells to produce prostaglandins. *Nature*, **268**, 447.

PACE-ASCIAK, C.R. & RANGARAJ, H. (1977). Distribution of prostaglandin biosynthetic pathways in several rat tissues. *Biochem. Biophys. Acta*, **486**, 579.

PLESCIA, O.J., SMITH, A.H. & GRINWICK, K. (1975). Subversion of immune system by tumor cells and role of prostaglandins. *Proc. Natl Acad. Sci. USA*, **72**, 1848.

PRENTICE, R.L., PEPE, M. & SELF, G. (1989). Dietary fat and breast cancer; a quantitative assessment of the epidemiological literature and a discussion of methodological issues. *Cancer Res.*, **49**, 3147.

SCHLAGER, S.I. & OHANIAN, S.H. (1979). A role for fatty acid composition of complex cellular lipids in the susceptibility of tumor cells to humoral immune killing. *J. Immunol.*, **123**, 146.

SCHLAGER, S.I. & OHANIAN, S.H. (1980*a*). Tumour cell lipid composition and sensitivity to humoral immune killing. I. Modification of cellular lipid and fatty acid content by metabolic inhibitors and hormones. *J. Immunol.*, **124**, 626.

SCHLAGER, S.I. & OHANIAN, S.H. (1980*b*). Tumour cell lipid composition and humoral immune killing. II. Influence of plasma membrane and intracellular lipid and fatty acid content. *J. Immunol.*, **125**, 508.

SOYDAN, A.S., YAZICI, Z., TAVARES, I.A., HOLLINGSWORTH, S. & BENNETT, A. (1991). Methotrexate alters the fatty acid profile of NC adenocarcinoma cells in culture. In *Eicosanoids and Other Bioactive Lipids in Cancer, Inflammation and Radiation Injury*, 2nd International Conference, Berlin, Abstr. p. 246.

TISDALE, M.J. & DHESI, J.K. (1990). Inhibition of weight loss by w-3 fatty acids in an experimental cachexia model. *Cancer Res.*, **50**, 5022.

WOOD, C.B., HABIB, N.A., THOMPSON, A. & 5 others (1985). Increase of oleic acid in erythrocytes associated with malignancies. *Br. Med. J. Clin. Res.*, **291**, 163.

YOUNG, M.R.I. & YOUNG, M.E. (1989). Effect of fish oil diets on prostaglandin-dependent and myelopoiesis-associated immune suppressor mechanism of mice bearing metastatic Lewis lung carcinoma tumors. *Cancer Res.*, **49**, 1931.